# The PAC-3 transcription factor critically regulates phenotype-associated genes in *Neurospora crassa*

**DOI:** 10.1590/1678-4685-GMB-2019-0374

**Published:** 2020-06-22

**Authors:** Maíra Pompeu Martins, Nilce Maria Martinez-Rossi, Pablo Rodrigo Sanches, Antonio Rossi

**Affiliations:** 1 Universidade de São Paulo, Faculdade de Medicina de Ribeirão Preto, Departamento de Genética, Ribeirão Preto, SP, Brazil

**Keywords:** RNA-seq, conidiation, hyphal development, transcription factor, inorganic phosphate

## Abstract

Transcription factors play an important role in fungal environmental adaptive process by promoting adjustment to challenging stimuli via gene modulation and activation of signaling networks. The transcription factor encoded by the *pac-3*/*rim101*/*pacC* gene is involved in pH regulation and is associated with a wide variety of cellular functions. The deletion of *pac-3* affects fungal development. In *Neurospora crassa*, the Δ*pac-3* strain presents diminished aerial growth and reduced conidiation. However, the PAC-3-regulated genes associated with this altered phenotype have not been elucidated. In this study, we used RNA-seq to analyze the phenotypic plasticity induced after *pac-3* deletion in the filamentous fungus *N. crassa* cultivated in media supplemented with sufficient or limited inorganic phosphate. Genes related to morphology, hyphal development, and conidiation were of particular interest in this study. Our results suggest a *pac-3* dependency in gene regulation in a Pi-dependent manner. Furthermore, our analysis suggested that the fungus attempts to overcome the deletion effects in a Δ*pac-3* mutant through a complex combined regulatory mechanism*.* Finally, the modulatory responses observed in the Δ*pac-3* strain, a double mutant generated based on the Δ*mus-52* mutant strain, is strain-specific, highlighting that the phenotypic impact may be attributed to *pac-3* absence despite the combined *mus-52* deletion.

## Introduction

To survive and proliferate, fungi must interact with and sense changes in their environment (Trevisan *et al.*, 2011). The adaptive success in different niches is resultant of the ability to scavenge for nutrients and respond to several challenging factors such as extreme temperature, carbon source, and pH changes (Han *et al.*, 1987; Bahn *et al.*, 2007; Martinez-Rossi *et al.*, 2017). Fungal sensing of the environment leads to the activation of intracellular signaling pathways, which are mediated by transcription factors. This network results in stress-associated transcriptional patterns which support the adaptation to specific stimuli (Braunsdorf *et al.*, 2016; Simaan *et al.*, 2019). The pH fitness of fungi is directly mediated by a C2H2 zinc finger transcription factor encoded by the *pac-3*/*rim101*/*pacC* gene, which is activated through the highly conserved Pal/Rim signaling pathway (Trevisan *et al.*, 2011; Rossi *et al.*, 2013). The pH signaling cascade activates gene regulation in response to acidic to alkaline pH shifts, which can extensively alter metabolic events (Rossi *et al.*, 2013).

Using mutant strains carrying the Δ*pac-3* (or *rim101*/*pacC*) revealed that the PAC-3 transcription factor correlates with fungal traits beyond pH signaling, impacting fundamental biological processes, including cell morphology, hyphae growth, conidiation, and adaptation to the host or nutritional variances (Ferreira-Nozawa *et al.*, 2006; Mendes *et al.*, 2012; O'Meara *et al.*, 2014; Martins *et al.*, 2018, 2019; Rascle *et al.*, 2018). In *Neurospora crassa*, the deletion of *pac-3* and the *pal* cascade-associated genes, except for Δ*pal-9*/*palI,* diminished aerial growth, reduced conidiation, and resulted in high production of melanin, in comparison with that in the wild-type strain (Virgilio *et al.*, 2016). A decrease in conidiation was also observed in the *Trichophyton interdigitale* H6 *pacC* mutant (Ferreira-Nozawa *et al.*, 2006). Additionally, the absence of *pacC* led to low conidiation in *Botrytis cinerea* (Rascle *et al.*, 2018), *Magnaporthe oryzae* (Landraud *et al.*, 2013), and *Aspergillus nidulans* (Tilburn *et al.*, 1995). Thus, there is a strong connection between the PAC-3 transcription factor and fungal development. However, the regulatory effect resultant of PAC-3 deletion on genes responsible for fungal development remains unexplored.

The expression of *pac-3*/*rim101*/*pacC* is modulated in response to environmental conditions, including variations in inorganic phosphate (Pi), carbon sources, and pH fluctuations, which may impair critical physiological functions (Ferreira-Nozawa *et al.*, 2006; Trevisan *et al.*, 2011; Mendes *et al.*, 2012; Martins *et al.*, 2019). In this study, we used RNA-seq analysis to assess the genes related to morphogenesis and development of the *N. crassa* mutant Δ*pac-3* cultivated in media containing sufficient or limited Pi, an essential constituent of biomolecules (Gras *et al.*, 2013). The purpose was to obtain evidence for transcriptional contribution to the observed phenotypical patterns. Pi is involved in diverse metabolic pathways and thus functions as a growth-limiting factor in microorganisms (Dick *et al.*, 2011; Vicent *et al.*, 2015). To determine whether *pac-*3-regulated genes are involved in the *N. crassa* phenotype, we studied the transcriptional impact that resulted from both the deletion and nutrient adaptive response simultaneously, as well as the changes that occurred exclusively due to the gene deletion independently of the nutritional condition. The results obtained provide evidence of the role of *pac-3*-mediated regulation in *N. crassa* growth and development, and indicate the genes possibly associated with the phenotypical effects observed in the Δ*pac-3* mutant.

## Material and Methods

### Culture conditions of *N. crassa* knockout strains

*N. crassa mus-52* ^KO^ (FGSC#9568) parental and Δ*pac-3* (NCU00090) knockout strains (Cupertino *et al.*, 2012) were maintained on solid Vogel's Minimal (VM) medium, pH 5.8 (Vogel, 1956) containing 2% sucrose at 30 °C. Approximately 10^7^ cells/mL^-1^ conidia were germinated in an orbital shaker for 5 h at 30 °C (200 rpm), as previously described (Martins *et al.*, 2019), in low- and high-Pi media (final concentrations, 10 μM or 10 mM Pi, respectively). The media was supplemented with 44 mM sucrose as the carbon source and adjusted to pH 5.4 with 50 mM sodium citrate (Nyc *et al.*, 1966; Gras *et al.*, 2007). The resulting mycelia was collected by filtering through 0.22-μm size filters (Millipore Corp., USA), frozen in liquid nitrogen, and stored at - 80 °C until RNA extraction. Experiments were performed in three biological replicates.

### RNA extraction, sequencing, data analysis, and functional enrichment

Total RNA was isolated using TRIzol Reagent (Invitrogen, USA) according to the manufacturer's instructions and treated with DNase I, RNase-free (Thermo Fisher, USA). The RNA concentration was quantified using a NanoDrop ND-1000 spectrophotometer (Thermo Fisher). The RNA integrity was determined by agarose formaldehyde gel electrophoresis and using the Agilent Bioanalyzer platform 2100 (Agilent, USA). Purity and concentration were measured using a NanoDrop ND-1000 spectrophotometer (Thermo Fisher). A total of 12 libraries (Δ*mus-52* and Δ*pac-3* strains, cultivated in high- and low-Pi concentration media, each, in three biological replicates) were sequenced on an Illumina HiSeq2000 (Illumina, USA) sequencer platform with paired-end 100-bp reads. RNA-seq data were analyzed and validated as previously described (Martins *et al.*, 2019) and deposited at the GEO database with accession number GSE132373.

### Selection of morphology- and development-related genes

After functional annotation analysis with the Blast2GO tool, the genes identified in the RNA-seq data were filtered with a customized R script, mapping modulated Genes and Gene Ontology (GO) terms. The script detected the descendant terms of the Gene Ontology nodes “cell wall,” “developmental process,” “cellular developmental process,” “regulation of biological process,” and “anatomical structure development” with the Bioconductor GO.db package (Carlson, 2018). We further identified morphology and development-related genes using literature data. The identified genes are listed in Table 1.

**Table 1:** Genes of *N. crassa* modulated in response to low-Pi or high-Pi concentrations. Comparisons were made for the double mutant Δ*pac-3* strain considering the Δ*mus-52* strain as control (Martins *et al.*, 2019) and for Δ*mus-52* strain considering the St.L.74-OR23-1VA strain as control (Martins *et al.*, 2018). The selected genes are associated with morphology and development regulation, including the Gene Ontology descendants search of the nodes “cell wall,” “developmental process,” “cellular developmental process,” “regulation of biological process,” and “anatomical structure development”.

GO ID	Gene ID	Gene Product Name	Δ*pac-3 vs.* Δ*mus-52*		Δ*mus-52 vs.* 74A	
			low-Pi	high-Pi	low-Pi	high-Pi
GO:0006355	NCU04058‡ †	BZIP domain-containing protein		3.00	2.77	
GO:0043666	NCU01504‡	Calcineurin binding protein		2.15		
GO:0070884						
GO:0006357	NCU08852‡	Poly(ADP-ribose) polymerase		2.18	2.66	
GO:0030435	NCU08726‡ †	Fluffy		1.94		
GO:0048315						
GO:0006357						
GO:0006355	NCU03650‡	DNA repair protein RAD16		1.55	1.77	
Unannotated	NCU04197‡	CipC protein	6.62	8.44		
GO:0006357	NCU00282‡ †	Zn(2)-C6 fungal-type domain-containing protein	3.89	4.34		
GO:0046830	NCU07723‡	Norsolorinic acid reductase	3.08	2.26		
GO:0006357	NCU04866‡ †	All development altered-6	2.00	2.15		
GO:0033499	NCU04442‡	GAL10	1.73	1.77		
GO:0000122	NCU08055‡ †	B-ZIP transcription factor IDI4	1.60	2.45		
GO:0001080						
GO:0045944						
GO:1903833						
GO:0007165	NCU06111‡	GTPase Ras2p	1.55	1.52		
Unannotated	NCU09629‡	Hypothetical protein	-1.81	2.12		-3.14
Unannotated	NCU04605‡	Hypothetical protein	-2.61			-3.39
GO:0043934	NCU00586‡	Non-anchored cell wall protein-6	-2.48			
GO:0006355	NCU00090‡ †	PH-response transcription factor pacC/RIM101	-1.80			
GO:0045013	NCU03965‡	Catabolite repression protein creC	-1.78			
GO:0072344	NCU03367‡	Hypothetical protein	-1.78			
GO:0005619	NCU08791‡	Catalase-1	-1.64			-2.12
GO:0048315						
GO:0061414	NCU02142‡ †	Zn(2)-C6 fungal-type domain-containing protein	-1.55		-4.62	-4.27
GO:0030968	NCU02235‡	Glycosyl hydrolase family 47-6		-6.03		
GO:0006355	NCU01931‡	Hypothetical protein		-1.98		
GO:0006357						
GO:0030435	NCU00399‡	Cell wall protein PhiA		-1.79		
GO:0007264	NCU02167‡	Krev-1-like		-1.57		
Unannotated	NCU01064‡	Hypothetical protein	-8.84	-5.77		
GO:1902600	NCU05046‡	E1-E2 ATPase-1	-6.77	-5.72		
GO:0055114	NCU04452‡	Menadione induced gene-3	-5.18	-5.77		
GO:1902600	NCU07966‡	Calcium-transporting ATPase 3	-4.00	-3.88		
GO:0009277	NCU07253‡	1,3-beta-glucanosyltransferase gel1	-3.56	-3.26		
GO:0016787	NCU07117‡	Ornithine-N5-oxygenase	-3.02	-2.28		
GO:0061414	NCU00155‡ †	C6 transcription factor	-2.10	-1.86		

Gene expression levels represented as log_2_-fold change comparing test and control strains in each of the Pi conditions (Δ*pac-3 vs.* Δ*mus-52* or Δ*mus-52 vs. N. crassa* St.L.74-OR23-1VA, identified as 74A strain in the table). Only values of log2FoldChange> = 1.5 or <= −1.5 were represented.

‡ Genes presenting binding motif for PAC-3 in their promoter region.

† Transcription factors

### *In silico* evaluation of the putative PAC-3-binding sites

The *N. crassa* OR74A genome (Ensembl Fungi) was used to search for the occurrence of the PAC-3 motif, 5'-GCCARG-3' (Tilburn *et al.*, 1995), in the 5' upstream regions (1 kb) of each gene (Table S1). The pursuit was determined using an *ad hoc* Perl script (Martins *et al.*, 2019).

## Results

### Global DEG identification and selection of genes associated with morphogenesis and development in the RNA-seq libraries

**Figure 1:**
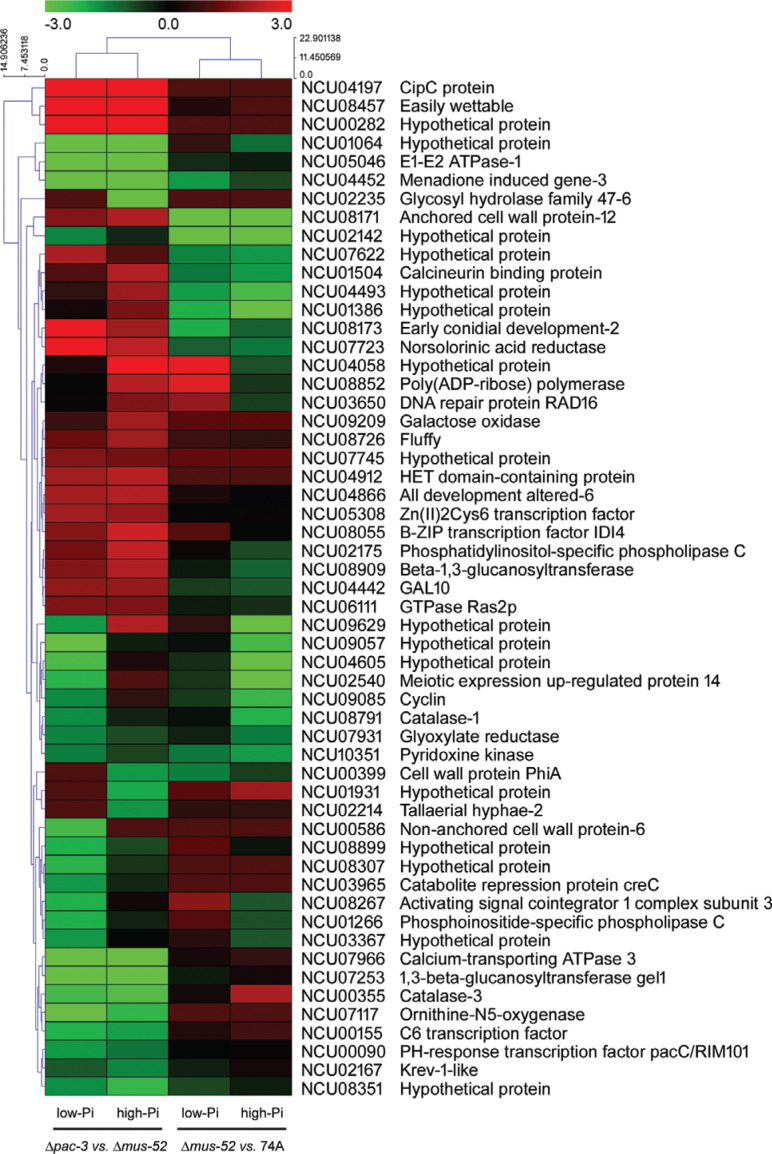
Heatmap of morphology and development regulation-associated genes from *N. crassa* mutants. The hierarchical clustering of expression patterns for the 55 differentially expressed genes identified in the Δ*pac-3*Δ*mus-52* mutant strain versus Δ*mus-52* is compared to the modulatory pattern observed for the same genes in the Δ*mus-52,* evaluated in comparison to the wild-type strain. Expression levels were loaded into the MultiExperiment Viewer (MeV) and analyzed using average-linkage hierarchical clustering with a Pearson correlation coefficient distance metric. The intensity of color represents the value of upregulation (red) or downregulation (green) in log2FC.

To perform a comprehensive analysis of the impact of Δ*pac-3* knockout on gene modulation, and to determine the effects of Pi variation on that response, we evaluated the results obtained by high-throughput sequencing (RNA-seq) using the *N. crassa mus-52* ^KO^ background strain as the control. By applying a cut-off threshold of at least 2.8-fold difference and a statistical significance threshold of P<0.05, 427 genes were identified as differentially modulated in response to both of the analyzed Pi conditions (Martins *et al.*, 2019). The results identified 55 genes that are associated with morphogenesis and development (Table 1). A heatmap of gene expression of the identified genes is shown in Figure 1. MultiExperiment Viewer (MeV) was used for hierarchical clustering by average linkage clustering based on Pearson correlation.

### Gene modulation in response to *pac-3* deletion

Among the identified genes that associated with the *N. crassa* developmental progress, catalase-3 (NCU00355), ornithine-N5-oxygenase (NCU07117), 1,3-beta-glucanosyltransferase *gel1* (NCU07253), two hypothetical proteins (NCU08351 and NCU01064), and two Ca^2+^-ATPases (E1-E2 ATPase-1, NCU05046; and calcium-transporting ATPase3, NCU07966) were repressed in both Pi conditions. Among the genes that were upregulated in response to *pac-3* deletion, regardless of the Pi condition, we identified CipC protein (NCU04197), GAL10 (NCU04442), GTPase Ras2p (NCU06111), and the anchored cell wall protein-12 (NCU08171).

### Gene modulation in response to Pi variation

The cell wall protein PhiA (NCU00399), fluffy (NCU08726), three hypothetical proteins identified as NCU09629, NCU04493, and NCU04605, a non-anchored cell wall protein-6 (NCU00586), and the galactose oxidase (NCU09209) were identified among the Pi-dependent genes.

## Discussion

Deciphering the regulatory mechanisms underlying the developmental programs in fungi can contribute to the elucidation of events that are unique and essential to these microorganisms. In this study, we aimed to identify the *pac-3*-regulated genes involved in *N. crassa* development in a transcriptional scenario, and thus contribute to the molecular understanding of the phenotype observed in the Δ*pac-3* mutant. Reduced growth and low conidiation were not observed in the parental background strain *mus-52* ^KO^, which indicated that the fungal development defects were due to the deletion of *pac-3*. Although *pac-3* deletion drastically affected the *N. crassa* phenotype, only a small number of genes associated with cell morphology, or hyphal and conidial development, were modulated in the evaluated conditions of Pi variance (Table 1). The phenotypic effect in the Δ*pac-3* strain may be the result of a more complex combined regulatory mechanism. Among the 55 genes that were identified, 31 possessed a binding motif for PAC-3 in their promoter regions (Table S1). The deletion of *pac-3* resulted in the differential modulation of 12 additional transcription factors, seven of which contained the PAC-3 binding motif.

### *N. crassa* gene downregulation is reflected in its phenotype

The transcriptional response that resulted explicitly from the absence of PAC-3, with Pi conditions being not a determinant of gene modulation, involved the downregulation of two catalase genes. Catalase-3 gene deletion provoked enhanced conidial production, hyphal adhesion, and more aerial hyphae in *N. crassa* (Michan *et al.*, 2003; Sun *et al.*, 2012), whereas catalase-1 deletion increased germination rate in *Metarhizium anisopliae* (Morales Hernandez *et al.*, 2010). Since catalase activity is protective against cellular component damage, the gene repression observed in this study may have led to oxidative stress toxic effects, which would impair *N. crassa* development.

Our results also identified that the ornithine-N5-oxygenase gene is downregulated. The encoded enzyme catalyzes the first step in the microbial-exclusive hydroxamate siderophore biosynthesis system (Eisendle *et al.*, 2003; Hissen *et al.*, 2005). Disruption of orthologue genes in *A. nidulans* and *Nomuraea rileyi* correlated the activity of the siderophore biosynthesis to decreased conidiation and defective hyphae elongation (Eisendle *et al.*, 2003; Li *et al.*, 2016).

Furthermore, 1,3-beta-glucanosyltransferase *gel1*, homologous to *gas1* in *Saccharomyces cerevisiae,* and *phr1* in *Candida albicans,* is downregulated*.* This gene encodes a GPI-anchored protein, required for correct morphogenesis and polar growth in these organisms (Ragni *et al.*, 2011). In *S. cerevisiae*, *gas1* deletion reduced fungi growth rate, and at an alkaline pH, the apical growth in the Δ*phr1* mutant was compromised in both yeast and hyphal growth forms (Mouyna *et al.*, 2000).

Among the downregulated hypothetical proteins, the gene identified as NCU08351 is directly associated with reduced conidial production, based on its knockout mutant strain (Sun *et al.*, 2012), and the gene identified as NCU01064 is related to the conidiation protein CON-6 (Suzuki *et al.*, 2013). The latter is a conidiation-specific time-dependent activator and is associated with the induction of *N. crassa* development (Bailey and Ebbole, 1998).

Two calcium-transporting pump genes, which are implicated in the maintenance of the proper level of calcium within cells, were repressed. The E1-E2 ATPase-1 and the calcium-transporting ATPase 3 are associated with hyphal morphogenesis in *N. crassa* (Silverman-Gavrila and Lew, 2001; Zelter *et al.*, 2004). In our results, morphology and development genes modulated in response to PAC-3 absence were highly repressed. The observed repression supported the observed reduced conidial production, in comparison with that in the wild-type strain. Further, the induction of other related genes suggested an attempt of the mutant fungi to overcome *pac-3* deletion.

### Δ*pac-3*-mutant attempts to restore developmental deregulation

We identified that CipC (from *c*oncanamycin-*i*nduced *p*rotein) protein was highly upregulated in both Pi conditions. This protein, exclusively found in fungi (Asif *et al.*, 2006), was associated with filamentous growth in *Ustilago maydis* (Rodriguez-Kessler *et al.*, 2012), and with hyphal development and conidial surface interactions in the pathogenic mold *Aspergillus fumigatus* (Asif *et al.*, 2006; Bauer *et al.*, 2010). In *A. nidulans*, the upregulation of CipC protein in a mutant strain deficient in glucosidase I could be one of the factors that contributed to the hyperbranching, resultant of its activity in polarizing growth (Zhang *et al.*, 2009).

The UDP-galactose-4-epimerase (GAL10), which was also upregulated, codes for an enzyme of the galactose metabolism. Its activity is associated with cell-wall integrity, morphology, and induces excessive filamentation in *C. albicans* (Singh *et al.*, 2007), and results in highly branched hyphae and reduced conidiation in *A. nidulans* (El-Ganiny *et al.*, 2010). We also identified the induction of the GTPase Ras2p, which codes for GTPase signal transducer proteins and acts as a regulator of growth and development (Fortwendel, 2015). In *N. crassa*, this protein is reported to be involved in the regulation of cell morphology, which affects the apical growth of hyphae and conidium formation (Kana-uchi *et al.*, 1997).

Proteomic analysis identified proteins that are secreted by the vegetative hyphae of *N. crassa* and expressed in a cell-type-specific manner (Maddi *et al.*, 2009)*.* One such protein, anchored cell wall protein-12, is implicated in cell wall remodeling and in the growth of the hyphae (Potapova, 2014). Our results revealed that the gene coding for this protein is induced in *N. crassa* in both Pi conditions. Our findings identified the overexpression of some developmental genes in the mutant strain, which indicates a possible attempt of the fungi to compensate for *pac-3* deletion.

### Δ*pac-3*-mutation is responsive to Pi variation

Among the genes presenting the *pac-3* binding motif, upon induction, the up-modulation occurred in high-Pi as a pattern. When repressed, with four exceptions (NCU02235, NCU01931, NCU00399, and NCU02167), the downregulation occurred in low Pi. Thus, our results suggest a *pac-3* dependency in gene regulation in a Pi-dependent manner.

The cell wall protein PhiA, an essential gene for conidia development and healthy growth in *A. nidulans* (Melin *et al.*, 2003), is repressed exclusively in high Pi conditions, suggesting that the Pi restriction directly impacts gene modulation. The Fluffy gene is induced in response to sufficient Pi. This gene codes for a C6 zinc finger transcription factor active in the regulation of two out of the five genes that act as specific regulators of conidiation in *N. crassa* (Mendes *et al.*, 2016)*.* Fluffy is expressed at a basal level in vegetative hyphae, and is transcriptionally activated in the aerial hyphae formation (Bailey and Ebbole, 1998; Rerngsamran *et al.*, 2005; Mendes *et al.*, 2016). The expression pattern of PhiA and Fluffy genes in high Pi suggests that Δ*pac-3* strain shows an attempt to restore conidiation by activating genes other than PhiA in the herein evaluated conditions.

Nineteen conidia-specific cell wall proteins were identified using a proteomic approach with *N. crassa* (Ao *et al.*, 2016). The promoter regions of each coding gene were analyzed to determine whether they were expressed in a conidia-specific manner (Ao *et al.*, 2016). Among these genes, four were modulated in our results. Two of them, identified as non-anchored cell wall protein-6 (NCU00586) and the hypothetical protein NCU04605 were downregulated in response to low-Pi. The other two, hypothetical protein NCU04493 and galactose oxidase (NCU09209), were induced in high-Pi. The two upregulated genes identified had conidiation-specific expression using the promoter approach, and the two downregulated genes, did not give a conidia-specific developmental expression pattern. These complementary results support the feasible role of the transcription factor *pac-3* in conidia-related regulation in high Pi.

The hypothetical protein NCU09629 presented with inverted modulation in Pi conditions. In limited Pi availability, this gene is repressed, and under sufficient Pi, it is induced. This gene shows the HET (for *het*erokaryon incompatibility [HI]) domain, a regulator of HI, and is associated with severe growth inhibition and negatively affects conidiation and hyphal compartmentation that leads to programmed cell death (Dementhon *et al.*, 2006).

### Genetic interactions render unexpected phenotype

The strain under analysis is a double mutant (Δ*pac-3*Δ*mus-52*). As described for negative genetic interactions (Mani *et al.*, 2008), the resultant phenotype was more substantial than expected. These genetic interactions frequently involve genes presenting leastwise partially overlapping functions, which may compensate for the deletion mutually (Hartman *et al.*, 2001). Growth-based gene interaction profiling is reasonable to subdivide the observed negative to positive interactions, although expression-based genetic interaction profiling provides a more specific understanding of the genetic interaction patterns (Amini *et al.*, 2019).

A heatmap that depicts the relative expression levels of the growth and development-associated genes identified in the Δ*pac-3*Δ*mus-52* mutant strain compared to the expression in the corresponding single mutant Δ*mus-52* is shown in Figure 1. As a pattern, the modulatory response observed in the single mutant was not sustained in the double mutant strain. Two profiles could be highlighted: in one of them, the modulatory pattern was strain-specific, as observed in the induced cluster including the gene identified as CipC protein (NCU04197) and the hypothetical protein NCU00282, or in the repressed cluster including the calcium-transporting ATPase 3 (NCU07966) and Krev-1-like (NCU02167) genes. In the second explicit expression profiling, the pattern skipped between high to low Pi in the different strains, as observed in a cluster, including the hypothetical protein NCU09629 and catalase-1 (NCU08791). In only a few genes, the expression pattern was maintained in both conditions and strains as for the pyridoxine kinase (NCU10351).

Our results provide evidence of the role of the double mutant-mediated regulation in *N. crassa.* In the same proportion that *mus-52* deletion incurs underestimated consequences to the organism (Martins *et al.*, 2018), the associated *pac-3* deletion reflects a pervasive and profound effect in *N. crassa* development, bringing relevant insights regarding biological networks.

## Supplementary Material

The following online material is available for this article:

Table S1 - Genes of *N. crassa* modulated in response to mutant strain Δ*pacC* (test) compared with the control strain (Δ*mus-52*) in medium containing low-Pi or high-Pi.
