# Supplementary material for: The PAC-3 transcription factor critically regulates phenotype-associated genes in Neurospora crassa
Source: Genet Mol Biol. 2020 Jun 22;43(3):e20190374. doi: 10.1590/1678-4685-GMB-2019-0374 (PMC7355564; doi:10.1590/1678-4685-GMB-2019-0374)
Supplement: Supplementary file 1 [file 1415-4757-GMB-43-3-e20190374-s1.pdf]

Genetics and Molecular Biology
https://doi.org/10.1590/1678-4685-GMB-2019-0374

Supplementary Material to "The PAC-3 transcription factor critically regulates phenotype-associated genes in *Neurospora crassa*"

Table S1 - Genes of *N. crassa* modulated in response to mutant strain Δ*pacC* (test) compared with the control strain (Δ*mus-52*) in medium containing low-Pi or high-Pi. These genes are associated with morphology and development regulation, including the Gene Ontology descendants search of the nodes "cell wall," "developmental process," "cellular developmental process," "regulation of biological process," and "anatomical structure development". The consensus binding sites were determined by identifying the following DNA sequences upstream (1,000bp) from the promoter region of the genes: PacC, 5'-GCCARG-3'.

Gene ID	Gene Product Name	Consensus number	Consensus	Positions
NCU04058	Hypothetical protein	1	GCCAGG	757
NCU01504	Calcineurin binding protein	1	CTTGGC (rc)	843
NCU08852	Poly(ADP-ribose) polymerase	4	CTTGGC (rc); CTTGGC (rc); CCTGGC (rc); CCTGGC (rc)	169; 875; 509; 924
NCU08726	Fluffy	3	CTTGGC (rc); CTTGGC (rc); CCTGGC (rc)	577; 607; 724
NCU03650	DNA repair protein RAD16	2	GCCAAG; GCCAAG	317; 840
NCU04197	CipC protein	1	GCCAGG	775
NCU00282	Hypothetical protein	1	CTTGGC (rc)	418
NCU07723	Norsolorinic acid reductase	1	GCCAAG	18
NCU04866	All development altered-6	1	CCTGGC (rc)	895
NCU04442	GAL10	1	CTTGGC (rc)	427
NCU08055	B-ZIP transcription factor IDI4	2	GCCAAG; GCCAGG	1; 110
NCU06111	GTPase Ras2p	1	GCCAAG	807
NCU09629	Hypothetical protein	1	CTTGGC (rc)	254
NCU04605	Hypothetical protein	1	CCTGGC (rc)	314
NCU00586	Non-anchored cell wall protein-6	2	CTTGGC (rc); CTTGGC (rc)	369; 573
NCU00090	PH-response transcription factor pacC/RIM101	5	CTTGGC (rc); CTTGGC (rc); CTTGGC (rc); CTTGGC (rc); CCTGGC (rc)	607; 618; 628; 867; 130

Gene ID	Gene Product Name	Consensus number	Consensus	Positions
NCU03965	Catabolite repression protein creC	1	CCTGGC (rc)	251
NCU03367	Hypothetical protein	2	CCTGGC (rc); CTTGGC (rc)	590; 922
NCU08791	Catalase-1	2	GCCAGG; GCCAGG	263; 632
NCU02142	Hypothetical protein	3	GCCAGG; GCCAAG; GCCAAG	409; 414; 605
NCU02235	Glycosyl hydrolase family 47-6	3	CTTGGC (rc); CTTGGC (rc); CTTGGC (rc)	700; 708; 747
NCU01931	Hypothetical protein	2	CCTGGC (rc); CTTGGC (rc)	507; 609
NCU00399	Cell wall protein PhiA	2	CCTGGC (rc); CTTGGC (rc)	181; 910
NCU02167	Krev-1-like	1	CTTGGC (rc)	523
NCU01064	Hypothetical protein	3	CTTGGC (rc); CTTGGC (rc); CTTGGC (rc)	217; 598; 745
NCU05046	E1-E2 ATPase-1	2	GCCAAG; GCCAAG	22; 31
NCU04452	Menadione induced gene-3	2	CTTGGC (rc); CTTGGC (rc)	845; 857
NCU07966	Calcium-transporting ATPase 3	2	GCCAAG; GCCAAG	265; 278
NCU07253	1,3-beta-glucanosyltransferase gel1	1	CTTGGC (rc)	735
NCU07117	Ornithine-N5-oxygenase	3	GCCAGG; GCCAGG; GCCAGG	419; 692; 887
NCU00155	C6 transcription factor	1	CTTGGC (rc)	263

DNA sequences upstream (1,000-bp)
rc: reverse complement
